# Hydroxychloroquine in the treatment of lichen planus-like cutaneous immune-related adverse events

**DOI:** 10.1016/j.jdcr.2025.10.002

**Published:** 2025-10-10

**Authors:** Kyle Mueller, Paul Bogner, Drew Kuraitis

**Affiliations:** a Jacobs School of Medicine and Biomedical Sciences, University of Buffalo, Buffalo, New York; b Department of Dermatology, Roswell Park Comprehensive Cancer Center, Buffalo, New York; c Department of Pathology, Roswell Park Comprehensive Cancer Center, Buffalo, New York; d Department of Dermatology, Tulane University, New Orleans, Louisiana

**Keywords:** cutaneous immune-related adverse event, hydroxychloroquine, immune checkpoint inhibitor, immunotherapy, lichen planus, lichenoid dermatitis

## Introduction

Use of immune checkpoint inhibitors (ICIs) allows for the stimulation of host T-cells to target cancers. Off-target effects are common and given the increasing use of ICIs for cancer therapy, dermatologists are increasingly likely to encounter patients with cutaneous toxicities, or cutaneous immune-related adverse events (cirAEs). In this context, roles of the dermatologist include correctly identifying the cirAE and adequately managing the eruption to facilitate the continuation of life-saving cancer therapies, and using steroid-sparing agents, if possible. Among the reported ICI-induced cirAEs, lichen planus-like (LPL)-cirAEs present with variable time of onset and are often managed with topical steroids, narrow-band UVB phototherapy, or systemic retinoids. In this paper, we present 2 cases of LPL-cirAEs successfully treated with hydroxychloroquine which salvaged ICI therapy without requiring systemic steroids. In the discussion, we review the literature on the use of hydroxychloroquine for managing LPL-cirAEs.

## Case 1

**Fig 1:**
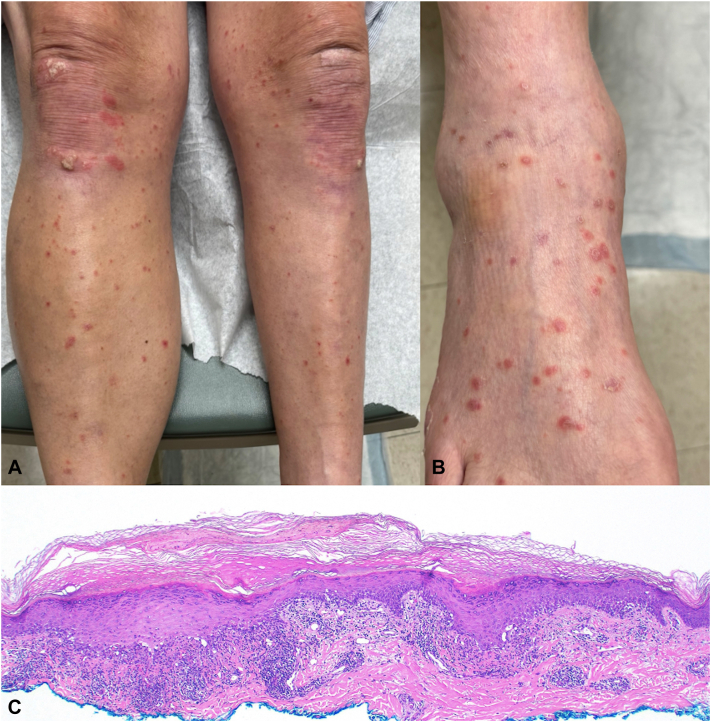
Initial presentation of Patient number 1 with erythematous scaly papules to the lower legs **(A)**, including the dorsal foot **(B)** after cycle 7 of pembrolizumab therapy. Histopathology of left dorsal foot lesion biopsy demonstrating patchy lichenoid damage with necrotic keratinocytes and dermal melanophages (**C,** 20×).

A 72-year-old man with a history of well-controlled plaque psoriasis and stage IIIc prostate adenocarcinoma receiving pembrolizumab therapy presented to dermatology for mildly pruritic lesions to his lower extremities that appeared after cycle 4 or 5 but worsened after cycle 7. There were erythematous scaling papules to the lower legs (Fig 1, *A*) and dorsal feet (Fig 1, *B*). There was no oral mucosal involvement. Biopsy of a representative lesion demonstrated patchy lichenoid damage to the epidermis with necrotic keratinocytes and dermal melanophages (Fig 1, *C*). Hepatitis B core antibody was negative, hepatitis C antibody was nonreactive and there was no peripheral eosinophilia. An LPL-cirAE was diagnosed. He was started on triamcinolone 0.1% cream twice daily.

**Fig 2:**
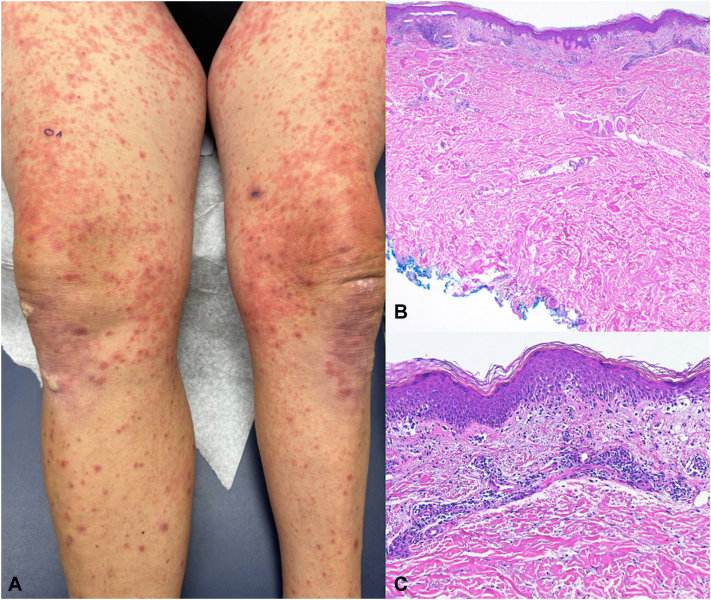
Patient number 1’s second and distinct eruption, considered to be a morbilliform eruption after amoxicillin exposure, and 1 week after presentation in Fig 1, demonstrating coalescing erythematous nonscaling papules to the legs **(A)** and trunk. Histopathology of morbilliform eruption demonstrating brisk perivascular dermal inflammation with eosinophils (**B,** 20×) and partially lichenoid interface dermatitis with epidermal dyskeratosis (**C,** 100×).

**Fig 3:**
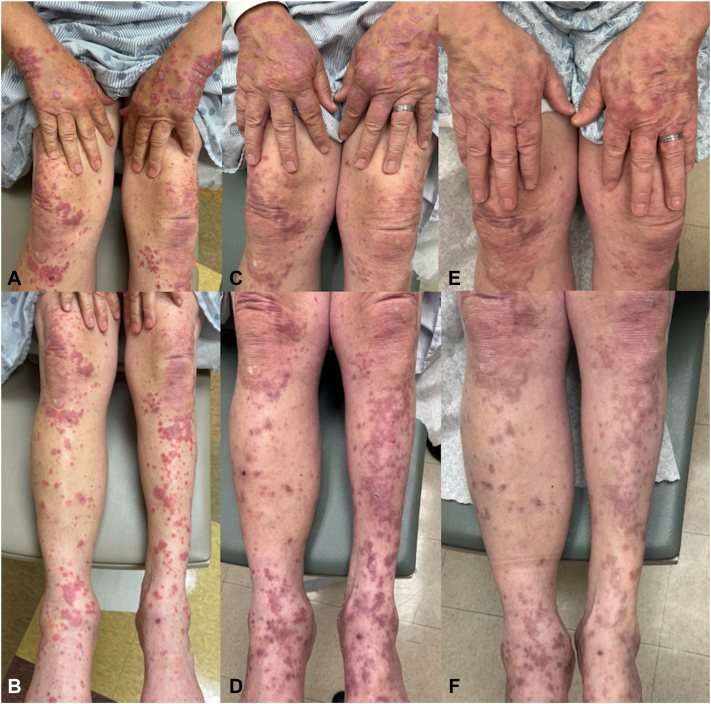
Evolving lichen planus-like cutaneous immune-related adverse event (cirAE) of Patient number 1, with violaceous papules coalescing into plaques, many with trailing scale to the hands, arms, and legs prior to starting hydroxychloroquine **(A** and **B)**. After 4 weeks of hydroxychloroquine, papules and plaques were much thinner and no longer scaling **(C** and **D)** in addition to having nearly resolved pruritus. Ten weeks later, residual erythematous and hyperpigmented macules and patches **(E** and **F)** remained at sites of prior lesions.

One week after his initial visit, the patient returned to the clinic with a new rash of 3 days’ duration. He started amoxicillin for a dental concern a few days prior. Examination found coalescing erythematous nonscaling papules to the thighs, abdomen, and back (Fig 2, *A*), sparing the oral mucosa. There was no lymphadenopathy. The patient was started on a prednisone taper, diphenhydramine, and continued triamcinolone for presumed superimposed amoxicillin-induced morbilliform eruption, and his pembrolizumab infusion was held for 1 week. Punch biopsy of the right thigh demonstrated brisk perivascular dermal inflammation with eosinophils and interface dermatitis with epidermal dyskeratosis, compatible with a drug eruption having some lichenoid interface changes (Fig 2, *B* and *C*). The rash responded quickly to prednisone, and he resumed pembrolizumab as expected. However, 8 weeks later, his initial cirAE recurred to the arms and legs with larger violaceous coalescing papules and plaques, many with trailing scale (Fig 3, *A* and *B*), and significant pruritus impairing qualify of life. His LPL-cirAE was diagnosed as grade 3 and hydroxychloroquine 200 mg twice daily was started. Four weeks later, his pruritus had nearly resolved, and his lesions had become much thinner (Fig 3, *C* and *D*). Ten weeks later, only postinflammatory erythema and hyperpigmentation remained (Fig 3, *E* and *F*). Pembrolizumab did not need to be held due to LPL-cirAE.

## Case 2

**Fig 4:**
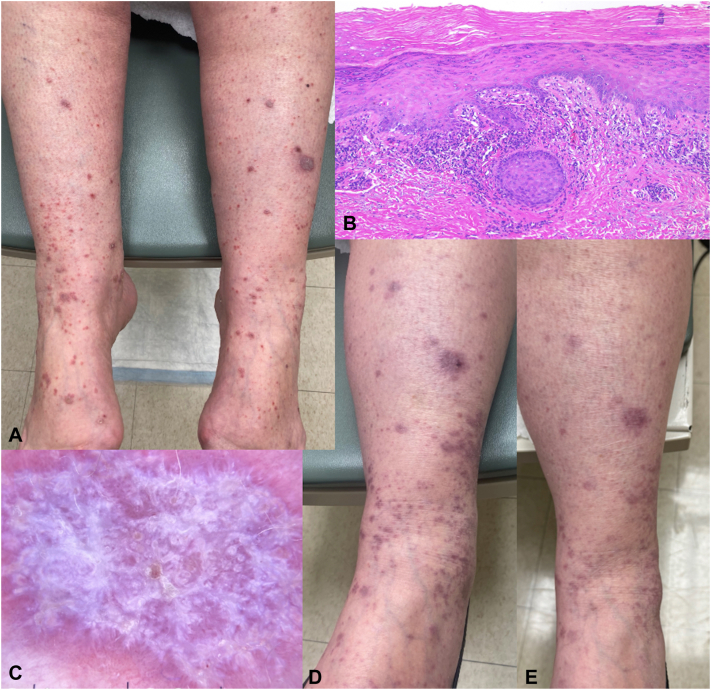
Initial presentation of Patient number 2 with violaceous scaly papules and plaques to the lower legs **(A)**. Histopathology demonstrated a brisk lichenoid band of subepidermal inflammation, damage to the basal keratinocytes and mild epidermal hyperplasia (**B,** 40×) and dermoscopy demonstrated *white dots* and irregular *white lines***(C)**. After 5 weeks of hydroxychloroquine, lesions had become much thinner and were no longer scaling on the left lower leg **(D)**, and only residual erythematous hyperpigmented macules and patches remained 16 weeks later **(E)**.

A 70-year-old woman undergoing palliative pembrolizumab for stage IIIc colon adenocarcinoma developed a pruritic rash 9 months after initiating therapy. The patient described a raised, itchy rash that began on the ankles and lower legs, then spread up the legs and to the arms and wrist, not responding to hydrocortisone. Physical examination found violaceous, slightly scaly papules, and thin plaques to the bilateral upper and lower extremities (Fig 4, *A*). There was no involvement of the oral mucosa. Hepatitis B core antibody was negative and hepatitis C antibody was nonreactive. There was a relative peripheral eosinophilia of 7.3%. A skin biopsy showed a brisk lichenoid band of subepidermal inflammation with lymphocytic infiltrate, damage to the basal keratinocytes, and mild epidermal hyperplasia (Fig 4, *B*) and dermoscopy demonstrated white dots and irregular white lines (Fig 4, *C*). A diagnosis of LPL-cirAE was made. Hydroxychloroquine 200 mg was started twice daily and the patient continued triamcinolone 0.1% cream as needed. She had rapid resolution in pruritus and 5 weeks later, her lesions had nearly resolved (Fig 4, *D*), with only postinflammatory changes remaining 16 weeks later (Fig 4, *E*). Throughout the treatment of the rash, the patient was able to continue pembrolizumab therapy without interruption.

## Discussion

Lichenoid eruptions comprise a common type of cirAE that carries significant morbidity, and lichenoid cirAEs secondary to immunotherapy are associated with treatment discontinuation and increased hospitalization rates.^1^ Goals of the oncodermatologist include managing cutaneous toxicities so that patients may stay on life-saving therapies if they are deriving clinical benefit. Despite the increased immunotherapy discontinuation rates and morbidity associated with lichenoid cirAEs, treatment guidelines are not yet standardized.^1^ While current management recommendations differ across different organizations, the National Comprehensive Cancer Network provides guidelines by grade of reaction^2^: for mild toxicity, ICI is continued, and high-potency topical corticosteroid or tacrolimus ointment can be used. For moderate toxicity, immunotherapy is held, high-dose prednisone may be started started until symptoms improve followed by a taper, and oral antihistamines or narrow-band UVB phototherapy may be considered. Severe toxicity recommendations include holding immunotherapy and administering prednisone or intravenous methylprednisone, with consideration for steroid-sparing immunosuppressants, such as acitretin.

Given that rashes frequently recur with subsequent ICI exposures, multiple iterations of rash management may be needed over the cancer treatment course. For patients with moderate or severe toxicity, management can entail repeated systemic steroid cycles and ultimately ICI discontinuation. However, long-term steroid usage may cause adverse effects in multiple organ systems and can reduce the efficacy of ICI therapy. Even in the short-term, an analysis of 6 clinical trials demonstrated that high peak doses of steroids for immune-related adverse events in patients on ICI therapy were associated with a significant decrease in progression-free and overall survival.^3^ Furthermore, high-dose short-term steroids can produce adverse effects in approximately one-third of patients.^4^ Therefore nonsteroidal, nonimmunosuppressant interventions, such as hydroxychloroquine, a disease-modifying antirheumatic drug, may represent a safer alternative for patients on ICI therapy,^5^ and could reduce the necessity for high-dose systemic steroids. To the best of our knowledge, 3 prior cases of LPL-cirAE reported successful management with treatment regimens including hydroxychloroquine.^6^, ^7^, ^8^ The immunomodulatory drug has been used in treating a wide range of dermatologic and rheumatologic conditions, with demonstrated efficacy in treating lichen planus.^9^ Its use in managing LPL-cirAEs has not yet been fully evaluated.

In line with the prior reports, treatment with hydroxychloroquine resulted in the resolution of our patients’ LPL-cirAEs. Our first case represented an unusual course, particularly with the acute morbilliform rash that elicited a hold on pembrolizumab for 1 week. Ultimately, the eruption represented hypersensitivity to amoxicillin, though we speculate that the partially lichenoid features on biopsy resulted from the patient’s ICI hypersensitivity-imposed inflammatory milieu.^10^ Interestingly, the patient did not experience worsening psoriasis, though it is a known cirAE of ICIs.^10^ For the second case, hydroxychloroquine was started soon after rendering a clinicopathologic diagnosis, resolving the rash quickly without requiring systemic steroid therapy or discontinuation of immunotherapy.

In 1 of the 3 previously reported cases, a 67-year-old woman with metastatic endometrial carcinoma developed painful oral erosions on the hard palate and mucosa and a pruritic, violaceous rash to her lower extremities, abdomen, and back 1 month after her first cycle of treatment with pembrolizumab and lenvatinib.^6^ Histopathology demonstrated a lichenoid interface dermatitis with a perivascular lymphoeosinophilic infiltrate. She was started on hydroxychloroquine 200 mg twice daily with tacrolimus swish and spit solution 3 times daily in the oral mucosa, and topical triamcinolone. One week later, after another pembrolizumab infusion, the rash worsened to include her flanks and extremities. While waiting for hydroxychloroquine response, pembrolizumab was temporarily held, and the patient received a prednisone taper. Two months later, the patient was clear of both oral and skin disease and resumed her ICI, which she tolerated with no recurrence of the lichenoid eruption.

A second prior case described a 78-year-old woman with stage II lung adenocarcinoma treated with pembrolizumab, who developed lesion to the extremities during her 6 months of therapy and up to 2 months after discontinuation.^7^ Some lesions were assumed squamous cell carcinoma at an outside clinic and received radiation therapy, after which multiple painful ulcers developed within previous lesions. Biopsies from various locations showed pseudoepitheliomatous hyperplasia with hyperkeratosis, hypergranulosis, and keratosis. After receiving a diagnosis of hypertrophic lichen planus, radiation was stopped and the patient received a prednisone taper, 200 mg hydroxychloroquine daily, and triamcinolone acetonide 0.1% ointment, with resolution of the lesions after 2 months.

In a third case, a 60-year-old man with metastatic renal cell carcinoma, initially treated with ipilimumab and nivolumab, developed a painless ulceration on the glans penis after cycle 16 of nivolumab monotherapy, or 32 weeks after initiation of treatment.^8^ The ulceration did not respond to oral or topical acyclovir for presumed herpetic infection. Biopsy revealed mucosal ulceration with a brisk, band-like lymphocytic infiltrate associated with vacuolar degeneration of basal keratinocytes. A diagnosis of nivolumab-induced lichenoid eruption was rendered, which resolved rapidly with triamcinolone 0.1% ointment. Subsequently, nivolumab was continued without interruption for another 3 months. Eight months into surveillance, the patient developed new oral lesions, recurrent penile ulcerations, and a pruritic rash to the extremities. He failed fluocinonide and high-potency topical corticosteroids for his oral lesions and rash, respectively. He was started on hydroxychloroquine 200 mg twice daily, with resolution in all lesions after approximately 3 weeks. These 3 prior cases and our 2 presented cases are summarized in Table I.

**Table I:** Clinicopathologic profile of patients with lichen planus-like cutaneous immune-related adverse events managed with hydroxychloroquine

Case report	Age and sex	Cancer type and stage	ICI type	Time to cirAE onset/ICI cycle #	Reported symptoms	Lesion location and mucosal involvement	Histopathology	History of HBV/HCV	Treatment to achieve remission of cirAE	ICI interruption, duration
Case 1	72 M	Prostate adenocarcinoma stage IIIc	Pembrolizumab	5 wk/C5 (1) + 8 wk/C10 (2)	Mild pruritus (1) Intense pruritus (2)	Dorsal forearms, LE (1) + UE (3); no mucosal involvement	Patchy lichenoid damage to the epidermis with necrotic keratinocytes and dermal melanophages	HBV (−) HCV (−)	Hydroxychloroquine, triamcinolone	Yes, 1 wk
Case 2	70 F	Colon adenocarcinoma stage IIIc	Pembrolizumab	9 mo/C13	Pruritus	LE, UE; no mucosal involvement	Brisk lichenoid band of subepidermal inflammation with lymphocytic infiltrate and damage to the basal keratinocytes and mild epidermal hyperplasia	HBV (−) HCV (−)	Hydroxychloroquine, triamcinolone	No
Alnajjar et al.^6^	67 F	Endometrial carcinoma stage IV	Pembrolizumab	1 mo/C1 (1) + 1 wk/C2 (2)	Pain, pruritus	LE, abdomen, back, oral mucosa (1) + flanks, UE (2)	Lichenoid interface dermatitis with a perivascular lymphoeosinophilic infiltrate	HBV (−) HCV (−)	Hydroxychloroquine, tacrolimus oral solution	Yes, 2 mo
Coscarart et al.^7^	78 F	Lung adenocarcinoma stage II	Nivolumab	<1 wk/C1	Asymptomatic	UE, LE; no mucosal involvement	Pseudoepitheliomatous hyperplasia with hyperkeratosis, hypergranulosis, and keratosis	NR	Prednisone taper, hydroxychloroquine, triamcinolone	No
Headd et al.^8^	60 M	Renal cell carcinoma stage IV	Pembrolizumab	32 wk/C16 (1) 18 mo/∗ (2)	Asymptomatic (1) pruritus (2)	Penis (1) + UE, LE, oral mucosa (2)	Mucosal ulceration with a brisk, band-like lymphocytic infiltrate associated with vacuolar degeneration of basal keratinocytes	NR	Triamcinolone (1) hydroxychloroquine (2)	No

*(1)*, Initial presenting symptoms; *(2)*, symptoms that developed after subsequent infusions or later in treatment course; *C[#]*, cycle number; *cirAE*, cutaneous immune-related adverse event; *F*, female; *HBV*, hepatitis B virus; *HCV*, hepatitis C virus; *ICI*, immune checkpoint inhibitor; *LE*, lower extremities; *M*, male; *NR*, not reported; *UE*, upper extremities.

∗ After ICI discontinuation.

Viewed together, these 5 cases demonstrate a diverse group of cancer types and LPL-cirAE presentations, including symptoms ranging from asymptomatic to intensely pruritic and with or without pain; variable morphologies; a wide variety of involved regions, with 2 of 5 involving mucosa; and unpredictable time to onset, ranging from within a week of therapy initiation to 8 months after discontinuing. Pembrolizumab, an anti-PD-1 monoclonal antibody, was the associated drug for 4 of the 5 cases. For the final treatment with hydroxychloroquine, topical triamcinolone was a common adjuvant. In 3 of 5 cases, ICI therapy did not need to be interrupted due to LPL-cirAE. The implication of these findings is limited, given the small sample size of cases. However, the success of hydroxychloroquine in these cases warrants investigation into its use for managing LPL-cirAEs. The safety of hydroxychloroquine with certain cancers, immunotherapies, and patient comorbidities would also require evaluation.

Through 2 case presentations and a literature review, we confirm the heterogeneity of LPL-cirAE clinical presentations, emphasizing the dermatologist’s role in monitoring patients receiving ICIs during treatment and after the discontinuation of ICI therapy. This role includes managing cutaneous toxicities so that life-saving therapy does not need to be discontinued, ideally also minimizing the burden of systemic steroid use. In practice, choosing hydroxychloroquine as a steroid-sparing agent involves assessing other options, such as acitretin, which similarly has reported efficacy in treating LPL-cirAEs.^11^ Both drugs are generally safe, however, acitretin requires additional laboratory monitoring and may present with more acute adverse events.^12^ Instead, hydroxychloroquine is well-tolerated, with fewer considerations for long-term use.^9^ With appropriate monitoring, hydroxychloroquine could be used for the duration of immunotherapy, and even for a time afterward, to prevent the recurrence of LPL-cirAE and facilitate uninterrupted cancer treatment. As such, hydroxychloroquine offers promise as a steroid-sparing agent for the management of LPL-cirAEs.

## Conflicts of interest

None disclosed.
